# Effects of receptor tyrosine kinase inhibitors on VEGF_165_a- and VEGF_165_b-stimulated gene transcription in HEK-293 cells expressing human VEGFR2

**DOI:** 10.1111/bph.13116

**Published:** 2015-04-10

**Authors:** Joanne J Carter, Amanda J Wheal, Stephen J Hill, Jeanette Woolard

**Affiliations:** Cell Signalling Research Group, School of Life Sciences, University of NottinghamNottingham, UK

## Abstract

**Background and Purpose:**

Receptor tyrosine kinase inhibitors (RTKIs) targeted at VEGF receptor 2 (VEGFR2) have proved to be attractive approaches to cancer therapy based on their ability to reduce angiogenesis. Here we have undertaken a quantitative analysis of the interaction of RTKIs and two VEGF splice variants, VEGF_165_a and VEGF_165_b, with VEGFR2 by studying nuclear factor of activated T-cells (NFAT) reporter gene activity in live HEK-293 cells.

**Experimental Approach:**

HEK-293 cells expressing the human VEGFR2 and a firefly luciferase reporter gene regulated by an NFAT response element were used for quantitative analysis of the effect of RTKIs on VEGF_165_a- and VEGF_165_b-stimulated luciferase gene expression.

**Key Results:**

VEGF_165_a produced a concentration-dependent activation of the NFAT-luciferase reporter gene in living cells that was inhibited in a non-competitive fashion by four different RTKIs (cediranib, pazopanib, sorafenib and vandetanib). The potency obtained for each RTKI from this analysis was similar to those obtained in binding studies using purified VEGFR2 kinase domains. VEGF_165_b was a lower-efficacy agonist of the NFAT-luciferase response when compared with VEGF_165_a. Analysis of the concentration–response data using the operational model of agonism indicated that both VEGF_165_ isoforms had similar affinity for VEGFR2.

**Conclusions and Implications:**

Quantitative pharmacological analysis of the interaction of VEGF_165_ isoforms and RTKIs with VEGFR2 in intact living cells has provided important insights into the relative affinity and efficacy of VEGF_165_a and VEGF_165_b for activation of the calcineurin- NFAT signalling pathway by this tyrosine kinase receptor.

## Tables of Links

**Table:** 

TARGETS
**Catalytic receptors**^*a*^
EGFR, EGF receptor
VEGFR1, VEGF receptor 1
VEGFR2, VEGF receptor 2
VEGFR3, VEGF receptor 3
**Enzymes**^*b*^
Akt
Abl1
MEK5
PKC
PLCγ

**Table:** 

LIGANDS
Cediranib
IP3, inositol-1,4,5-trisphosphate
Pazopanib
Sorafenib
Vandetanib
VEGF-A
VEGF-B
VEGF-C
VEGF-D

These Tables list key protein targets and ligands in this article which are hyperlinked to corresponding entries in http://www.guidetopharmacology.org, the common portal for data from the IUPHAR/BPS Guide to PHARMACOLOGY (Pawson *et al*., 2014) and are permanently archived in the Concise Guide to PHARMACOLOGY 2013/14 (*^a,b^*Alexander *et al*., 2013a,b[Bibr b1],[Bibr b2]).

## Introduction

VEGF is an important mediator of cell survival, proliferation and angiogenesis (Ferrara, 2009; Shibuya, 2011; Musumeci *et al*., 2012). It constitutes a family of mammalian homodimeric glycoproteins, comprising VEGF-A, VEGF-B, VEGF-C, VEGF-D and placenta growth factor. VEGF-A is an important and potent mediator of tumour-induced angiogenesis (Ferrara, 2004; 2009[Bibr b11],[Bibr b12]). VEGF family members bind to three different VEGF receptors (VEGFR1, VEGFR2 and VEGFR3) with differing selectivity profiles (Ferrara, 2009; Shibuya, 2011). VEGFR2 is the major regulator of VEGF-driven responses in vascular endothelial cells including permeability, proliferation, invasion and migration. It is also considered to be a crucial mediator of angiogenesis (Ferrara, 2009; Shibuya, 2011). Its signalling pathways are relatively well understood with tyrosine residues Y1175 and Y1214 in the human VEGFR2 being the main auto-phosphorylation sites activated by VEGF binding and tyrosine kinase activation. This creates binding sites for key intracellular signalling proteins such as Grb2, PLCγ and Shc1 (Matsumoto and Mugishima, 2006; Rososki, 2008; Koch *et al*., 2011).

The transmembrane glycoprotein neuropilin 1 forms a complex with VEGFR2 and acts as a co-receptor to enhance VEGF-A binding, mediate focal adhesion kinase phosphorylation and increase cell migration (Herzog *et al*., 2011). It has also been shown to promote VEGFR2 internalization and endosomal trafficking, leading to the regulation of ERK signalling and cell proliferation (Lanahan *et al*., 2013). Neuropilin 1 is engaged by specific VEGF isoforms and has recently been the target of drug discovery efforts to design low MW inhibitors of neuropilin 1 (Djordjevic and Driscoll, 2013). Multiple isoforms of VEGF-A, ranging from 121 to 206 amino acids, can be generated by alternative exon splicing that differ in their ability to bind heparin (affecting bioavailability) or neuropilin 1 and they appear to play distinctive roles in angiogenesis (Woolard *et al*., 2004; 2009[Bibr b30],[Bibr b31]; Ferrara, 2009). For example, alternative splicing in exon 8 of the VEGF gene can generate VEGF_xxx_a and VEGF_xxx_b (where xxx is the amino acid length) isoforms that have been reported to have pro-angiogenic and anti-angiogenic activities respectively (Woolard *et al*., 2004; 2009[Bibr b30],[Bibr b31]). In keeping with this, VEGF_165_b has been reported to be a weak partial agonist at VEGFR2, able to bind weakly to heparin and does not interact with neuropilin-1 (Cebe Suarez *et al*., 2006; Catena *et al*., 2010).

Receptor tyrosine kinases inhibitors (RTKIs) targeted at VEGFR2 have proved to be attractive approaches to cancer therapy based on their ability to reduce angiogenesis and/or lymph-angiogenesis (Musumeci *et al*., 2012). There are three known classes of RKTIs. Class I RTKIs, such as cediranib, vandetanib and pazopanib, are able to bind to the active conformation of the receptor and compete for the intracellular ATP-binding site within the catalytic domain of VEGFR2 (Gotink and Verheul, 2010; Davis *et al*., 2011; Blasi *et al*., 2012). Class II RTKIs, such as sorafenib, bind to the non-active conformation of the receptor at the hydrophobic pocket of the activation loop and inhibit kinase activity by indirectly preventing the binding of ATP (Gotink and Verheul, 2010; Davis *et al*., 2011). Finally, class III RTKIs such as neratinib, covalently bind to cysteine residues within the intracellular ATP-binding region of the receptor (Gotink and Verheul, 2010; Davis *et al*., 2011). Most of these small molecule RTKIs interact with multiple members of the PK family (Davis *et al*., 2011). For example, binding studies with purified kinase domains have shown that vandetanib is a more potent inhibitor of Abl1 (16 nM), EGFR (9.5 nM), MEK5 (49 nM) than VEGFR2 (820 nM) (Davis *et al*., 2011).

Quantitative evaluation of the interactions of RTKIs with VEGFR2 in living cells has, however, been largely lacking. This is important as, by definition, all RTKIs need to access the intracellular regions of VEGFR2 in order to elicit their pharmacological action. It is therefore vital to understand how the different RTKIs affect VEGF_165_a- and VEGF_165_b-mediated signalling in intact cells. The aim of the present study was to undertake a quantitative pharmacological analysis of the effect of VEGF_165_ isoforms and RTKIs on VEGFR2-mediated signalling in living cells. An important signalling pathway for VEGFR2 is the calcineurin-nuclear factor of activated T-cells (NFAT) system that, following activation by VEGF, leads to nuclear translocation of the NFAT transcription factor and expression of pro-angiogenic and pro-inflammatory genes (Suehiro *et al*., 2014; Yang *et al*., 2014). Reporter gene systems have been used extensively to study GPCRs and provide an alternative to biochemical assays for following signal transduction pathways from receptors at the cell surface to nuclear gene transcription in living cells (Hill *et al*., 2001). Here we have used an NFAT-luciferase reporter gene to investigate the impact of four representative RTKIs on VEGF_165_a- and VEGF_165_b-stimulated NFAT-luciferase activity in HEK-293 cells expressing human VEGFR2.

## Methods

### Cell lines

HEK-293 cells expressing the human VEGFR2 and an NFAT reporter gene were provided by Promega Corporation. The NFAT reporter gene contained an NFAT response element linked via a minimal promoter to the firefly luciferase gene luc2P containing a human sequence enriched in proline (P), glutamic acid (E), serine (S) and threonine (T) protein destabilization sequence (Voon *et al*., 2005). VEGFR2 NFAT cells were maintained in DMEM media supplemented with 10% FCS and 0.5% G418 in a humidified 5% CO_2_/95% air atmosphere at 37°C.

### Measurement of VEGFR2-stimulated NFAT-reporter gene activity in HEK-293 cells

VEGFR2 NFAT cells were seeded in a T75 flask at 5 × 10^6^ cells per flask using DMEM +10%FCS and incubated at 37°C in a 5% CO_2_/95% air atmosphere for 3 days until the cells were 100% confluent. On the fourth day, cells were washed with PBS and detached using 3 mL Versene® (ETDA 0.02% in PBS). Once cells had detached, 6 mL of DMEM +0.1%BSA was added and the cells were counted using a haemocytometer. Cells were centrifuged at 200× *g* for 5 min, resuspended in DMEM +0.1%BSA and seeded at a density of 4 × 10^4^ cells per well in 80 μL DMEM +0.1%BSA in white-sided, clear flat-bottomed 96-well plates (Greiner, Stonehouse, UK), which had been coated with 0.01 mg·mL^−1^ poly-D-lysine in PBS for 30 min and washed with DMEM. Cells were then incubated for 1 h in a humidified 5% CO_2_/95% air atmosphere at 37°C. RTKIs or vehicle control were added in 10 μL DMEM +0.1%BSA for 1 h prior to addition of VEGF_165_a or VEGF_165_b in 10 μL DMEM +0.1%BSA and the incubation was continued for a further 5 h (in a humidified 5% CO_2_/95% air atmosphere at 37°C). After the 5 h incubation, 100 μL ONE-Glo Luciferase Assay reagent was added to each well and luminescence was measured according to the manufacturer's instructions on a Topcount platereader (Perkin Elmer, Llantrisant, UK).

### Data analysis

All data were fitted using non-linear regression in Prism 6 (GraphPad Software, San Diego, CA, USA). VEGF_165_a and VEGF_165_b concentration–response curves were fitted to the following equation:

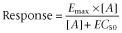
1

Where *E*_max_ is the maximal response, and the EC_50_ is the molar concentration of agonist required to generate 50% of the *E*_max_. When investigating the effect of different concentrations of RTKI on concentration–response curves for VEGF_165_a, the data were also fitted to Equation 2013 with parameters for either EC_50_ of *E*_max_ shared between all curves. A comparison of the extra sum of squares that resulted from the analysis with separate EC_50_ or *E*_max_ values (over that with one of the parameters shared) using the *F*-test (Prism 6) then allowed for statistical analysis of the difference between EC_50_ or *E*_max_ values.

Inhibition curves obtained with RTKIs in the presence of a fixed concentration of VEGF_165_a or VEGF_165_b were fitted to the following equation:

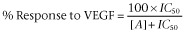
2

Where [*A*] is the concentration of RTKI and the *IC*_50_ is the molar concentration of ligand required to inhibit 50% of the response to VEGF.

Partial agonist concentration–response curves to VEGF_165_b were also fitted to the operational model of Black and Leff (1983) using the following equation:

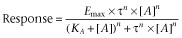
3

Where *E*_max_ is the maximal response of the system, [*A*] is the concentration of VEGF_165_b, *n* is the slope parameter, *K*_A_ is the dissociation constant of the agonist VEGF_165_b and τ is the transducer constant, which is a practical measure of efficacy. τ is the inverse of the fraction of receptors that must be occupied by agonist to obtain the half-maximal response. *E*_max_ was determined by simultaneously fitting Equation 2013 to the concentration–response data for VEGF_165_a that were obtained in the same experiments as those for VEGF_165_b. *E*_max_ was shared between the two simultaneous fits.

Equation 1983 was also used to simultaneously fit concentration–response curves to VEGF_165_a in the presence and absence of increasing concentrations of a define RTKI. In this case, *E*_max_, *n* and *K*_A_ (which in this case is the dissociation constant of VEGF_165_a) were shared between the simultaneous fits.

All data are presented as mean ± SEM. The *n* in the text refers to the number of separate experiments. Statistical significance was determined by Student's unpaired *t*-test or by one or two-way anova with Dunnett's *post hoc* analysis and *P* < 0.05 was considered statistically significant.

### Materials

VEGF_165_a and VEGF_165_b were obtained from R&D systems (Abingdon, UK). Vandetanib, pazopanib, cediranib and sorafenib were supplied by Sequoia Research Products (Pangbourne, UK). The ONE-Glo™ Luciferase Assay System was obtained from Promega Corporation (Madison, WI, USA). Versene was obtained from Lonza (Basal, Switzerland). G418 was purchased from Life Technologies (Paisley, UK). All other chemicals and reagents were purchased from Sigma-Aldrich (Gillingham, UK).

## Results

### VEGF_165_a-stimulated NFAT-luciferase production in intact cells

**Figure 1:**
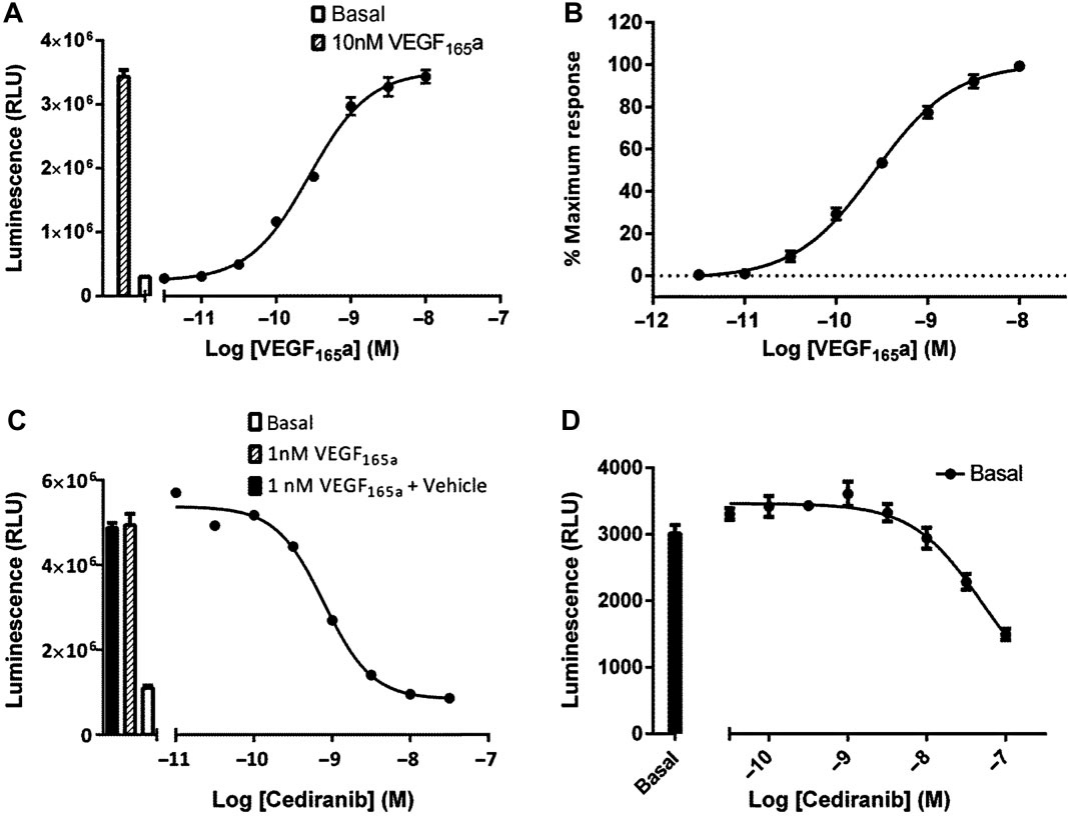
The effect VEGF_165_a on NFAT-mediated gene transcription in VEGFR2 NFAT cells. VEGFR2 NFAT cells were treated with VEGF_165_a (A and B) or cediranib +1 nM VEGF_165_a (C). Data are mean ± SEM from quadruplicate determinations in a single representative experiment that was repeated on five separate occasions (A and C). Normalized data from five repeat experiments expressed as a percentage of the response to 10 nM VEGF_165_a in each experiment (B). Effect of cediranib on basal NFAT-luciferase activity (D). Data are mean ± SEM from quadruplicate determinations in a single representative experiment that was repeated on five separate occasions. The histogram in (A) and (C) show the control response to 1 nM VEGF_165_a (A and C) and that to VEGF_165_a in the presence of the vehicle (containing DMSO) for the highest concentration of cediranib used in the competition experiment shown in (C).

**Figure 2:**
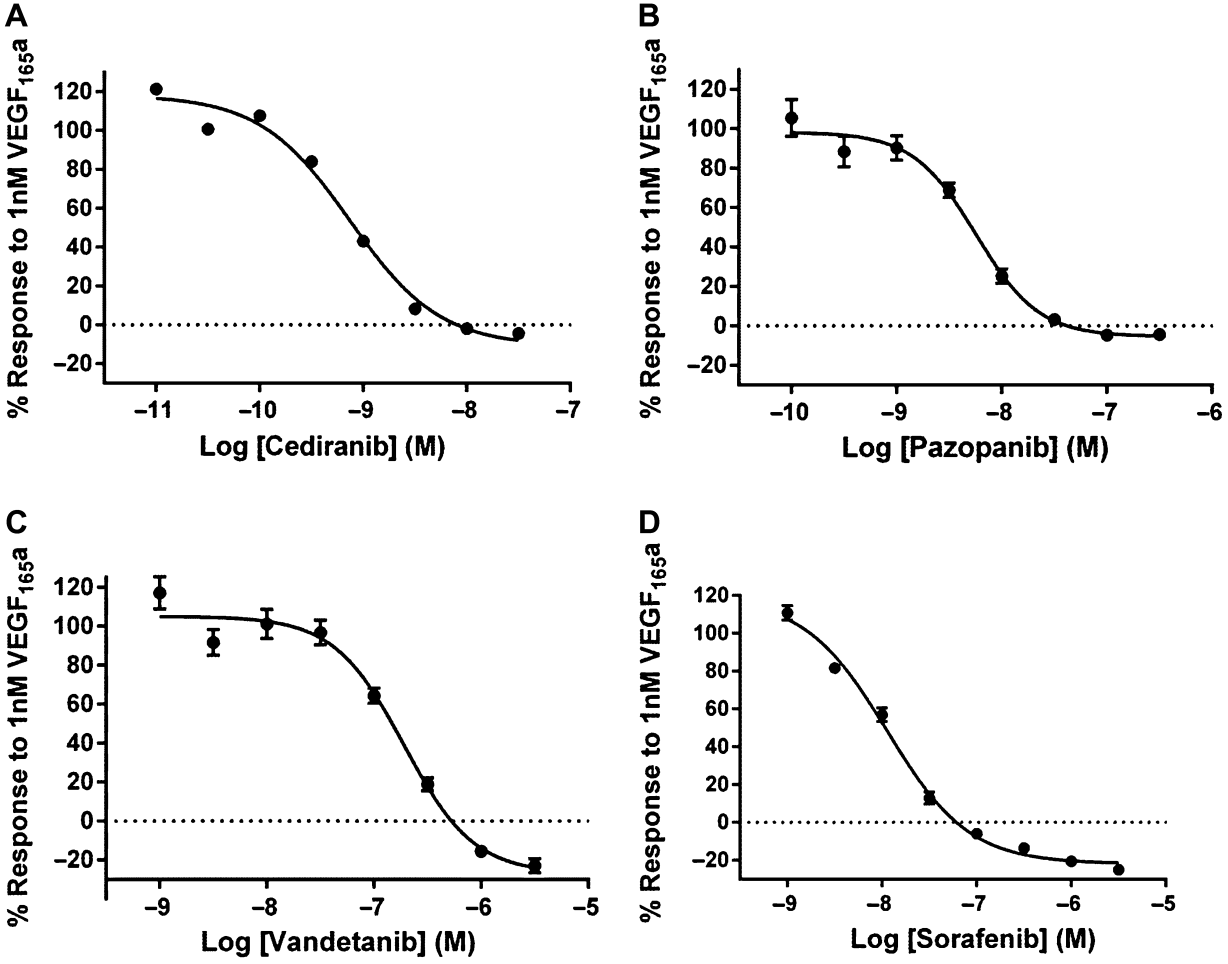
The effect of selected RTKIs on NFAT gene transcription stimulated by 1 nM VEGF_165_a. VEGFR2 NFAT cells were treated with (A) cediranib, (B) pazopanib, (C) vandetanib or (D) sorafenib. Data are mean ± SEM of five separate experiments. Data are expressed as a percentage of the response to 1 nM VEGF_165_a in the absence of RTKIs. Each individual experiment was performed in quadruplicate.

Incubation with VEGF_165_a produced a concentration-dependent (pEC_50_ 9.66 ± 0.05, *n* = 10) increase in NFAT-mediated luciferase production in HEK-293 cells expressing VEGFR2 that was 8.30 ± 0.85-fold (*n* = 10) over basal levels (Table 1; Figure 1A and B). The response to 1 nM VEGF_165_a was inhibited by the RTKI cediranib in intact HEK-293 cells in a concentration-dependent manner (Figure 1C; Table 2). The pIC_50_ obtained for cediranib (9.13; Figure 2A, Table 2) was in close agreement with that reported from binding studies with the purified VEGFR2 kinase domain (Davis *et al*., 2011). It was also noticeable that there was no marked inhibition by cediranib below basal levels at the highest concentration used (Figures 1C and 2A), suggesting that the ability of this RTKI to inhibit other tyrosine kinases (e.g. PDGFR-A, PDGFR-B and EGFR, Davis *et al*., 2011) did not significantly impact on the response observed. This was confirmed when the effect of cediranib was evaluated for its ability to inhibit basal NFAT-luciferase production (Figure 1D). A significant inhibition (*P* < 0.05; one way anova) of the small basal NFAT-luciferase response was only observed at concentrations of cediranib above 10 nM (Figure 1D). Analysis of all five repeat experiments indicated that a significant inhibition of basal signalling was only obtained at the two highest concentrations used (*P* < 0.05; one way anova; *n* = 5).

**Table 1:** Concentration–response parameters for VEGF_165_a- and VEGF_165_b-stimulated NFAT-luciferase responses

	−Log EC_50_	*E*_max_ (% VEGF_165a_ max)	*n*
VEGF_165_a	9.66 ± 0.05	100	10
VEGF_165_b	9.21 ± 0.08	62.1 ± 1.2	5

Values are mean ± SEM of *n* separate experiments. Each individual experiment was performed in quadruplicate.

**Table 2:** The effect of selected RTKIs on VEGF-stimulated firefly luciferase production in VEGFR2 NFAT cells

	Inhibition of 1 nM VEGF_165_a pIC_50_	*n*	Inhibition of 3 nM VEGF_165_b pIC_50_	n	Reported binding pK_D_ for purified kinase domain*
Cediranib	9.13 ± 0.01	5	9.38 ± 0.07	5	8.96
Pazopanib	8.25 ± 0.03	5	8.29 ± 0.10	8	7.85
Sorafenib	8.01 ± 0.06	5	7.96 ± 0.04	5	7.23
Vandetanib	6.72 ± 0.03	5	7.00 ± 0.04	6	6.08

VEGFR2 NFAT cells were treated with each RTKI and either 1 nM VEGF_165_a or 3 nM VEGF_165_b. Data are mean ± SEM of *n* separate experiments. Each individual experiment was performed in quadruplicate. Individual fitted values for pIC_50_ values were obtained in each individual experiment and then analysed to provide mean ± SEM data provided here.

* Values taken from Davis *et al*., 2011.

Inhibition of 1 nM VEGF_165_a-stimulated NFAT-luciferase activity was also obtained with a second-class I RTKI (pazopanib, which has a different inhibitor selectivity profile compared with cediranib, e.g. FGFR1-3, PDGFRA/B, VEGFR1 and EGF; Davis *et al*., 2011) and with sorafenib and vandetanib (Figure 2, Table 2). All RTKIs tested produced pIC_50_ values, which were in agreement with those reported previously in binding studies on purified VEGFR2 kinase domains (Table 2). As with cediranib, there was no marked inhibition below basal responses (Figure 2B) with pazopanib (which is also a potent PDGFR inhibitor, Davis *et al*., 2011). In contrast, both sorafenib (−25.0 ± 2.6%, *n* = 5) and vandetanib (−23.0 ± 3.7%, *n* = 5) produced a small significant inhibition (*P* < 0.05, paired *t*-test) below basal levels, which may reflect some interference with other tyrosine kinases at the higher concentrations required to inhibit VEGFR2 with these inhibitors.

**Figure 3:**
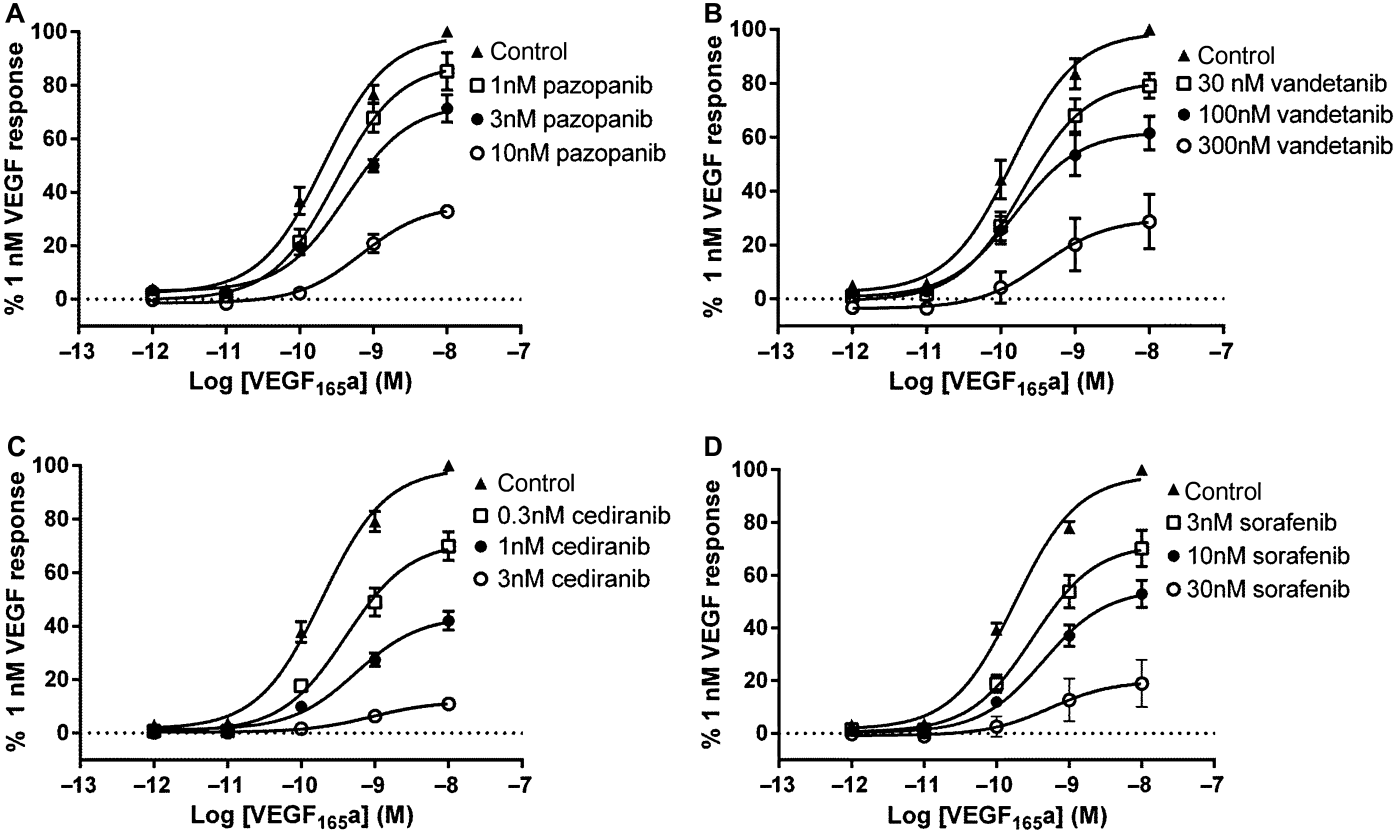
The effect of RTKIs on VEGF_165_a concentration–response curves. VEGFR2 NFAT cells were treated with (A) pazopanib, (B) vandetanib, (C) cediranib or (D) sorafenib for 1 h prior to the addition of increasing concentrations of VEGF_165_a. Data are mean ± SEM of five (A and B), six (C) or seven (D) replicate experiments. Each individual experiment was performed in quadruplicate. Global analysis of the combined data presented for each RTKI (A–D; extra sum of squares *F*-test) indicated that there was only a significant difference in the EC_50_ values for cetiranib (*P* < 0.05). In contrast, there was a significant decrease in *E*_max_ with all four RTKIs (*P* < 0001; Figure 3; extra sum of squares *F*-test).

VEGF binding to VEGFR2 requires Ig-like domains D2 and D3 in the extracellular portion of the receptor (Dosch and Ballmer-Hofer, 2010; Leppänen *et al*., 2010). In contrast, the kinase domain, which is the target for RTKIs lies within the intracellular portion of the receptor. As a consequence, the interaction between VEGF and RTKI in intact cells should show classical non-competitive interactions when concentration–response curves to VEGF_165_a are analysed in the presence of increasing concentrations of RTKIs. These data for VEGF-stimulated NFAT-luciferase production are shown in Figure 3 and Table 3. All four inhibitors produced a significant (*P* < 0.05) concentration-dependent reduction in the maximal response to VEGF_165_a (Table 3). Analysis of all the individual experiments indicated that there was a small, but significant change (*P* < 0.05) in EC_50_ at the highest concentrations of RTKIs used (Table 3). However, global analysis of the combined data presented in Figure 3 indicated that there was only a significant difference in the EC_50_ values for cediranib (*P* < 0.05). In contrast, there was a significant decrease in *E*_max_ with all four RTKIs (*P* < 0.001; extra sum of squares *F*-test; Figure 3).

**Table 3:** Effect of RTKIs on VEGF_165_a concentration–response parameters

Vandetanib			Pazopanib			Cediranib			Sorafenib		
nM	pEC_50_	% *E*_max_	nM	pEC_50_	% *E*_max_	nM	pEC_50_	% *E*_max_	nM	pEC_50_	% *E*_max_
0	9.90 ± 0.14	100.0	0	9.66 ± 0.11	100.0	0	9.68 ± 0.09	100.0	0	9.72 ± 0.06	100.0
30	9.77 ± 0.10	81.0 ± 4.1	1	9.50 ± 0.09	85.2 ± 7.0	0.3	9.40 ± 0.10	64.8 ± 10.4*	3	9.50 ± 0.05	70.1 ± 6.9*
100	9.75 ± 0.12	58.5 ± 5.7*	3	9.43 ± 0.13	71.4 ± 5.2*	1	9.28 ± 0.12	31.1 ± 6.6*	10	9.33 ± 0.06*	52.9 ± 5.1*
300	9.41 ± 0.14*	29.5 ± 7.9*	10	9.14 ± 0.16*	32.8 ± 2.0*	3	9.13 ± 0.18*	8.8 ± 1.9*	30	9.00 ± 0.13*	18.9 ± 8.9*

pEC_50_ and *E*_max_ values for VEGF_165_a obtained in the presence of increasing concentrations of four RTKIs.

* *P* < 0.05 compared with corresponding control in the absence of RTKI (one-way anova). Values are mean ± SEM from six (cediranib), five (pazopanib), seven (sorafenib) and five (vandetanib) separate experiments.

### Pharmacological characteristics of the splice variant, VEGF_165_b, in HEK-293 cells

**Figure 4:**
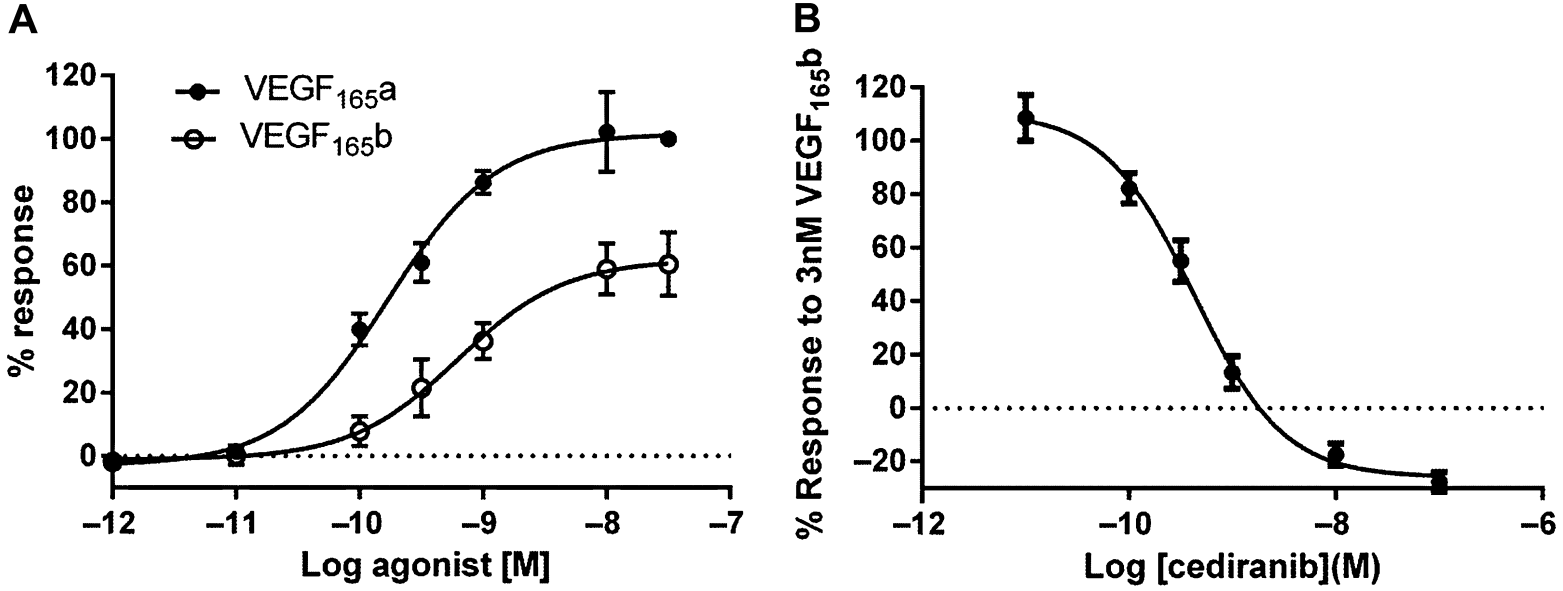
Characterization of the effect of VEGF_165_b on NFAT-luciferase repsonses. (A) A comparison of VEGF_165_a and VEGF_165_b concentration–response curves. (B) Inhibition of VEGF_165_b-stimulated NFAT-luciferase responses by cediranib. In (B), the concentration of VEGF_165_b was 3 nM. Data are mean ± SEM of five separate experiments. Four replicates were made for each condition in each individual experiment.

In the present study, VEGF_165_b produced a robust NFAT-luciferase response in HEK-293 cells expressing human VEGFR2 that accounted for 62.1% (Table 1, Figure 4) of the maximum response obtained with VEGF_165_a in the same experiments. The EC_50_ values were, however, very similar (Table 1, Figure 4A). Analysis of the concentration–response curves using the operational model of Black and Leff (1983) for partial agonists indicated that the log *K*_A_ for VEGF_165_b was −8.83 ± 0.13 (*n* = 5) and the transducer constant **τ** was 1.65 ± 0.23 (*n* = 5). **τ** is a measure of agonist efficacy and represents the inverse of the fraction of receptors (60.1%) that must be occupied by agonist to obtain the half-maximal response (Black and Leff, 1983). The response to 3 nM VEGF_165_b was sensitive to inhibition by RTKIs with similar potencies to those obtained when VEGF_165_a was used as agonist (Figure 4B; Table 2).

## Discussion

VEGF receptors have been shown to activate several intracellular signalling pathways including PKC, PLCγ, MAPK and calcium-calcineurin (Suehiro *et al*., 2014). Calcineurin signalling activates NFAT transcription factors leading to the stimulation of gene transcription (Hill *et al*., 2001; Suehiro *et al*., 2014; Yang *et al*., 2014). Stimulation of PLCγ increases levels of inositol-1,4,5-trisphosphate (IP_3_) and diacyglycerol. IP_3_ then stimulates the release of intracellular calcium while diacylglycerol activates PKC. Increased intracellular calcium concentration stimulates calcineurin leading to the dephosphorylation of cytoplasmic NFAT transcription factors allowing them to translocate to the nucleus. In parallel, PKC activation results in the production of the AP-1 immediate early genes c-fos and c-jun. Once in the nucleus, NFAT binds with c-fos and c-jun to form a transcriptional complex capable of synergistically activating both the NFAT and the AP-1 response elements to stimulate gene expression (Masuda *et al*., 1998; Macian *et al*., 2000; Hill *et al*., 2001). In endothelial cells, VEGF treatment leads to NFAT nuclear localization and the expression of pro-angiogenic and pro-inflammatory genes (Suehiro *et al*., 2014; Yang *et al*., 2014). Furthermore, the calcineurin-NFAT pathway appears to be an important route for VEGF-mediated signalling (Suehiro *et al*., 2014; Yang *et al*., 2014). Here we have used a reporter gene containing the NFAT promoter coupled to the expression of firefly luciferase (Hill *et al*., 2001; Voon *et al*., 2005) to investigate in living cells the pharmacological characteristics of VEGF_165_a- and VEGF_165_b-induced gene expression in HEK-293 cell transfected with human VEGFR2.

Both VEGF_165_a and VEGF_165_b were able to produce a robust and potent stimulation of NFAT-mediated luciferase gene expression after 5 h of incubation. Both isoforms had very similar EC_50_ values that were in the nanomolar range (Table 1). This is in keeping with previous reports that VEGF_165_b has a lower efficacy than VEGF_165_a for VEGFR2-mediated responses (Woolard *et al*., 2004; Cebe Suarez *et al*., 2006; Kawamura *et al*., 2008; Catena *et al*., 2010). The alternatively spliced variant VEGF_165_b was a partial agonist of NFAT-luciferase production eliciting a maximal response that was only 62% of that achieved by VEGF_165_a. Analysis of the VEGF_165_b concentration–response data using the operational model of Black and Leff (1983) provides a means by which both the dissociation binding constant (*K*_A_) and the efficacy (in terms of the τ constant) of VEGF_165_b can be estimated. This produced an estimate for pK_A_ of VEGF_165_b at VEGFR2 (8.83) and a value of 1.65 for the efficacy parameter τ (indicating that 60.1% of receptors need to be occupied by VEGF_165_b in order to achieve 50% of the maximal cellular response). The similarity of EC_50_ values for the two VEGF isoforms, however, suggests that the relatively large response to VEGF_165_b compared with that seen in other studies (Cebe Suarez *et al*., 2006; Kawamura *et al*., 2008; Catena *et al*., 2010) is not a consequence of a large amplification of the signalling pathways to NFAT-mediated gene expression in these cells. Furthermore, previous work has suggested that VEGF_165_a and VEGF_165_b have the same binding affinities for VEGFR2 (Woolard *et al*., 2004; Cebe Suarez *et al*., 2006).

Interestingly, previous work has indicated that the extent to which VEGF_165_b can elicit responses may depend on the cellular context and the signalling cascade measured. Thus, while VEGF_165_b was a very weak agonist of MAPK and Akt phosphorylation in VEGFR2 transfected CHO cells, it was able to stimulate a robust MAPK and Akt phosphorylation in human microvascular endothelial cells (Woolard *et al*., 2004). Furthermore, signalling pathway differences in the relative efficacy of VEGF-A isoforms have been observed for the activation of VEGFR2-mediated responses (ERK1/2, p38 MAPK, Akt) in HUVECs by VEGF_165_a and VEGF_121_a (Fearnley *et al*., 2014). Previous studies, however, have largely been based on Western blot analysis, and it is clear that the NFAT-luciferase system reported here provides a powerful system for the quantitative evaluation of concentration–response relationships for drugs interacting with human VEGFR2 in living cells.

Four representative RTKIs (cediranib, pazopanib, vandetanib and sorafenib) were able to inhibit both VEGF_165_a- and VEGF_165_b-mediated NFAT-luciferase expression with similar potency, and yielded IC_50_ values that were similar to the *K*_D_ values reported from binding studies with purified VEGFR2 kinase domains (Davis *et al*., 2011). This similarity suggests that all four compounds readily cross the cell membrane in intact living cells. The target for VEGF binding within VEGFR2 is to domains D2 and D3 of the extracellular portion of the receptor (Dosch and Ballmer-Hofer, 2010; Leppänen *et al*., 2010). In contrast, RTKIs interact in various ways (depending on RTKI class) with the intracellular kinase domain of the receptor. As a consequence, the interaction between VEGF and RTKI in intact cells would be expected to show classical non-competitive interactions and lead to a marked change in the maximum response to VEGF_165_a with little impact on the EC_50_ value for the agonist. This is what was observed in the present study (Figure 3). All four RTKIs produced a marked reduction in the *E*_max_ values for VEGF_165_a with only a small change in the pEC_50_ value. At the highest concentrations of RTKIs used, the pEC_50_ was generally between 9.00 and 9.14, which provides an indication of the PK_A_ for VEGF_165_a. Global analysis of the combined data shown in Figure 3 for each inhibitor (using the extra sum of squares *F*-test) indicated that it was only cediranib that had a significant difference in EC_50_ between the four sets of VEGF_165_a concentration–responses curves.

The non-competitive nature of the inhibition produced by RTKIs via the intracellular kinase domain, however, provided an opportunity to estimate the binding affinity of VEGF_165a_ by utilizing the operational model of Black and Leff (1983). In this model, the transducer ratio tau (τ) is a measure of efficacy and reflects the ratio (total receptor number)/*K*_E_ where K_E_ describes the hyperbolic relationship in the system between the response and the concentration of agonist-receptor complexes. Receptor alkylation experiments have been used previously to reduce the number of binding sites as a way to obtain concentration–response relationships with different τ values, but with common values for *K*_E_, *E*_max_ and *K*_A_. Use of an RTKI targeted against VEGFR2 kinase activity is also a way of reducing τ values without changing the maximal capacity of the NFAT reporter gene system in the cells. In this case, the inhibition of the VEGFR2 kinase activity will interfere with signal transduction at the level of the receptor. This will effectively alter the *K*_E_ value and increase the concentration of agonist–receptor complexes needed to produce a function response. However, this analysis assumes that the RTKI has no allosteric effect on the binding affinity of VEGF_165_a. Thus, increasing concentrations of RTKIs should decrease the apparent efficacy of VEGF_165_a in this cellular system and reduce the transducer constant τ. All other parameters in terms of *K*_A_, *E*_max_ and should, however, remain unchanged.

If the concentration–response curves obtained with a given RTKI presented in Figure 3 are simultaneously analysed on this basis (with these assumptions), the following estimates of the pK_A_ value for VEGF_165_a are obtained: 8.9, 8.9, 9.4 and 9.0 (for the data sets obtained with cediranib, sorafenib, vandetanib and pazopanib respectively). The mean value obtained from this analysis for VEGF_165_a (8.9) is almost identical to that obtained for the partial agonist VEGF_165_b (8.8) in keeping with previous reports that their affinities are identical (Woolard *et al*., 2004; Cebe Suarez *et al*., 2006). Interestingly, vandetanib produced a higher estimate for the pK_A_ of VEGF_165_a than that obtained with the other RTKIs. This suggests that the nature of the interaction of vandetanib with VEGFR2 has produced an allosteric conformational change in the ligand binding site for VEGF_165_a and altered its affinity for VEGF_165_a.

It should be noted that all four RTKIs produced a small inhibition of the responses to VEGF_165_a and VEGF_165_b below basal levels (Figure 2; Figure 4B). A similar effect has been seen with other RTKs (Forsell *et al*., 2012) and has been ascribed to constitutive activity of the receptor. However, in the present study, the effect is likely to be due to inhibition of other tyrosine kinases within this cell line. The effect of cediranib on basal NFAT signalling seen in Figure 1D is consistent with this, particularly at the higher concentration used. Thus, the potency of cediranib for inhibition of VEGF-stimulated NFAT signalling and basal NFAT signalling, respectively, are quite different (Figure 1C and D). Thus, although the major effects of RTKIs reported here are consequences of an interaction with VEGFR2, it must be remembered that interference with other tyrosine kinase signalling cascades is possible at higher concentrations of these inhibitors.

In summary, the present study has shown that the VEGFR2 NFAT-luciferase reporter gene system provides a robust way to investigate, in a quantitative manner, the interaction of drugs (both agonists and RTKIs) with VEGFR2 in an intact cellular environment. Quantitative pharmacological analysis of the interaction of these drugs with VEGFR2 in living cells has provided important insights into the relative affinity and efficacy of VEGF_165_a and VEGF_165_b for activation of the calcineurin–NFAT signalling pathway by this tyrosine kinase receptor. This opens the way for similar quantitative approaches to be used to evaluate affinity and efficacy measures for different VEGF isoforms in mediating responses via other signalling cascades. This should shed light on the potential for VEGFR2 agonists to bias signalling to particular intracellular pathways.

## Abbreviations

NFAT: nuclear factor of activated T-cells
RTKIs: receptor tyrosine kinase inhibitors
VEGFR1: VEGF receptor 1
VEGFR2: VEGF receptor 2
VEGFR3: VEGF receptor 3
